# Epigenetics of Dendritic Cells in Tumor Immunology

**DOI:** 10.3390/cancers14051179

**Published:** 2022-02-24

**Authors:** Gerard Godoy-Tena, Esteban Ballestar

**Affiliations:** 1 Epigenetics and Immune Disease Group, Josep Carreras Research Institute (IJC), 08916 Barcelona, Spain; ggodoy@carrerasresearch.org; 2 Epigenetics in Inflammatory and Metabolic Diseases Laboratory, Health Science Center (HSC), East China Normal University (ECNU), Shanghai 200241, China

**Keywords:** dendritic cells, tumor microenvironment, epigenetics, DNA methylation, tolerogenesis, cancer epigenetics, histone modification, tumor immunology

## Abstract

**Simple Summary:**

Dendritic cells (DCs) are an important type of immune cell present in the blood and tissues, capable of detecting potential threats and displaying them to lymphocytes in the lymph nodes, therefore initiating lymphocyte-mediated responses. DCs not only recognize pathogens but also damaged or altered cells from our own bodies, such as cancer cells, and contribute to the immune response to cancer. However, the tumor microenvironment, the environment that surrounds cancer cells, produces a number of factors that can modulate the function of DCs, which can acquire an immunosuppressive phenotype that allows tumor growth. This acquisition is also tightly regulated by epigenetics, the set of mechanisms that impact gene function without altering the DNA sequence. In this review, we discuss epigenetic mechanisms that influence the development of functional DCs and their altered function in the tumor microenvironment. We propose how this knowledge can be used both to epigenetically modulate these cells, and for the development of DC-based vaccine therapies.

**Abstract:**

Dendritic cells (DCs) are professional antigen-presenting cells with the distinctive property of inducing the priming and differentiation of naïve CD4+ and CD8+ T cells into helper and cytotoxic effector T cells to develop efficient tumor-immune responses. DCs display pathogenic and tumorigenic antigens on their surface through major histocompatibility complexes to directly influence the differentiation of T cells. Cells in the tumor microenvironment (TME), including cancer cells and other immune-infiltrated cells, can lead DCs to acquire an immune-tolerogenic phenotype that facilitates tumor progression. Epigenetic alterations contribute to cancer development, not only by directly affecting cancer cells, but also by their fundamental role in the differentiation of DCs that acquire a tolerogenic phenotype that, in turn, suppresses T cell-mediated responses. In this review, we focus on the epigenetic regulation of DCs that have infiltrated the TME and discuss how knowledge of the epigenetic control of DCs can be used to improve DC-based vaccines for cancer immunotherapy.

## 1. Introduction

Efficient immune responses to threats involve a wide range of cell types within the adaptive and innate immune systems. These threats include pathogens, self-antigens in autoimmune conditions, and damaged or aberrant cells in cancer. Cancer cells display aberrant phenotypes and behavior as genetic mutations and epigenetic alterations accumulate, resulting in malignant transformation of cells [1].

To achieve a correct immune response to cancer cells, it is critical that antigen-presenting cells (APCs) activate T cells by cross-presenting antigens from the tumor. Dendritic cells (DCs) are specialized APCs that are needed to stimulate T cell-driven anticancer immunity and make it more robust. Recent studies have expanded our knowledge about the role of the different DC subtypes in the immune antitumor response. However, in most cases, tumor-infiltrating DCs develop an immune-tolerant phenotype that favors tumor growth [2,3]. This is in part due to the effects that cancer cells exert on different immune cell types through cell-to-cell contacts, secretion of soluble factors and exosome release, which influence their epigenetic and gene expression profiles. In recent years, the tumor microenvironment (TME), i.e., the environment around a tumor, including the surrounding blood vessels, the infiltrating immune cells, fibroblasts, and extracellular matrix, has been recognized as a major player in tumor progression.

Epigenetic dysregulation provides several mechanisms for cancer development and progression due to its effects on the aberrant activation of oncogenes and repression of tumor suppressor genes. Epigenetics is also relevant in this context as it links extracellular signals with changes in gene expression. Most cancer epigenetic studies have focused on cancer cells, which display aberrant changes in DNA methylation and histone modifications. However, epigenetic alterations are involucrate in numerous processes in the DC development and recruitment and might also play a crucial role in the switch from an immunogenic to a tolerogenic phenotype in DCs. The potential role of epigenetic dysregulation in tumor-infiltrated DCs is also highly relevant as a result of the potential pharmacological reversion of epigenetic alterations and immunogenic phenotype, as well as in the context of using these mechanisms for improving DC-based vaccines.

## 2. Mechanisms Underlying Epigenetic Dysregulation

Epigenetic mechanisms, in conjunction with transcription factors (TFs), define the transcriptional status of different gene sets, and those that are transcribed in a given cell type and in a particular situation ultimately determine cell function. In cancer cells, epigenetic alterations profoundly disrupt their transcriptome. In relation to DCs, it is well established that epigenetics plays a crucial role in the control of immune cell differentiation, identity, and function. The role of epigenetics in the differentiation of the myeloid lineage, which is particularly relevant to most DC subtypes, has been thoroughly described [4,5]. In addition, recent studies have revealed strong connections between epigenetics and cytokine production by tumor cells [6].

Epigenetic alterations mainly involve changes in DNA methylation, histone modifications and non-coding RNAs. Methylation of the 5′ position of cytosines (5meC) followed by guanines (CpG dinucleotides) is the best studied epigenetic modification. This chemical modification can influence the binding and recruitment of numerous proteins, including TFs and chromatin-modifying regulators. DNA methylation is catalyzed by DNA methyltransferases (DNMTs). These enzymes are classified in two main types: maintenance DNMTs, such as DNMT1, which are responsible for copying the DNA methylation patterns from the parental DNA strands during DNA replication; and de novo DNMTs, such as DNMT3A and DNMT3B [7]. The removal of methyl groups can be passive, by the inefficient action of DNMT1 during replication, or active. Active DNA demethylation is mediated by the concerted activity of the enzymes of the ten-eleven translocation (TET) family, thymine DNA glycosylase (TDG), and the base-excision repair (BER) machinery [8]. Alterations in the methylation patterns of DNA are responsible for cell differentiation, and their dysregulation leads to diseases, including cancer [9].

Histone post-translational modifications are the second main group of epigenetic marks. They may be associated with transcriptional activation or repression. There are several types of post-translational modification, including acetylation of Lys, methylation of Lys and Arg, phosphorylation of Ser and Thr, and others. These chemical modifications not only have direct effects on chromatin structure, but also facilitate the binding of other factors. Different enzymes are responsible for the deposition and removal of these post-translational modifications, including histone acetyltransferases (HATs), histone deacetylases (HDACs), histone methyltransferases (HMTs), and histone demethylases (HDMs), among others. The nature of the chemical modification and the position of the amino acid residue in the protein sequence yield different functional outcomes. Generally, histone H3 trimethylation at Lys9 (H3K9me3) and Lys27 (H3K27me3) are considered repressive modifications [10], whereas histone acetylation at the same positions (H3K9ac and H3K27ac) in promoters and enhancers, respectively, are generally associated with transcriptional activation, and the removal of these acetyl marks is linked to gene repression. Conversely, H3 trimethylation at Lys4 (H3K4me3) and Lys36 (H3K36me3) are also associated with active transcription [10].

Non-coding RNAs (ncRNAs) are also considered a type of epigenetic mechanism, although, in this case, it does not involve any chemical modification. Specifically, it consists of RNA molecules that do not encode proteins, yet have functions after being spliced and/or processed into smaller products. ncRNAs include microRNAs (miRNAs), small nucleolar RNAs (snoRNAs) as well as tens of thousands of longer transcripts, such as long non-coding RNAs (lncRNAs), most of the functions of which are still unknown [11]. For instance, miRNAs primarily act at a post-transcriptional level by base-pairing with their complimentary mRNA targets to alter mRNA stability and/or translation into protein [12]. mRNA destabilization explains the vast majority of miRNA-mediated repression [13].

## 3. Dendritic Cell Subtypes and Epigenetic Regulation

DCs are professional APCs with the distinctive property of inducing priming and differentiation of naïve T cells like CD4+ and CD8+ T cells into helper and cytotoxic effector T cells, respectively. This role has been confirmed by the study of DC-deficient animals, obtained by, for instance, knocking out Batf3, an essential TF for several DC subtypes [14,15]. DCs mature following the detection of pathogen-associated molecular patterns (PAMPs) from viruses and bacteria, and of damage-associated molecular patterns (DMAPs), in the case of cancer cells, through binding to pattern recognition receptors such as Toll-like receptors (TLRs). During maturation, major histocompatibility complex (MHC) molecules move to the DC plasma membrane, upregulation of CD80/CD86 molecules and IL-12 is produced [16]. This maturation can also be accompanied by loss of DNA methylation followed by de novo methylation. This has been attributed to modulation of the expression of DNMT1, DNMT3A, and DNMT3B [17]. DC maturation is also accompanied by changes in histone modifications. A recent study has demonstrated that the intracellular heat shock protein 70-like protein (HSP70L1) inhibits human DC maturation. Intracellular HSP70L1 inhibits the recruitment of Ash1l and maintains repressive H3K27me3 and H2AK119Ub1 modifications on the promoter regions of the MHC and STAT3 genes [18]. Another study has shown that Polycomb group factor 6 (PCGF6) is associated with the H3K4me3 demethylase JARID1c, and together, they negatively regulate H3K4me3 levels in DCs, which are necessary for DC activation [19].

DCs are not only required to stimulate T cell-driven anticancer immunity, but also to make it more robust. Antigens from tumor cells are internalized and loaded through MHC molecules. This process activates DCs that then migrate towards draining lymph nodes, where they can induce an adaptive immune response by presenting these antigens to T cells [20]. Antigen presentation by DCs occurs through the T-cell receptor (TCR), which is expressed in naïve T cells and can recognize these antigens. Once the TCR-MHC interaction has occurred, T cells initiate the process of activation and differentiation, known as T-cell priming. During this process, there is also a global remodeling of the epigenome, including changes in DNA methylation [21,22] and histone modification [23,24] in T cells. Subsequently, primed T cells migrate to the tumor, where they exert cytotoxic anti-tumor effects.

One of the characteristics that makes DCs so important is their ability to present antigens by both MHC-I and MHC-II molecules [25,26]. The unconventional presentation of antigens loaded onto MHCs by DCs relies on ‘‘cross-presentation’’ [27], which is needed to ensure immunity to viruses, intracellular bacteria, and cancer cells. Antigens loaded onto MHC-II molecules can be recognized by antigen-specific CD4+ T helper cells. In addition, MHC-I molecules can be recognized by antigen-specific CD8+ T cells, leading to their proliferation and the activation of their cytotoxic capabilities [27].

Phenotypic and functional criteria have customarily been used to define three main DC subtypes, namely, conventional DCs (cDCs), plasmacytoid DCs (pDCs) and monocyte-derived DCs (moDCs). cDCs, characterized by the expression of CD11c, derive from common DC precursors (CDPs) in the bone marrow (BM). cDCs can be further split into two main lineages: cDC1 and cDC2. cDC1s have an enhanced ability to cross-present exogenous antigens on MHC-I and to activate CD8+ T cells. This subtype is characterized by the expression of CD141 in humans [28,29] and XCR1 in mice. In comparison, cDC2s, characterized by the expression of CD1c in humans [26] and CD11b in mice, represent a heterogeneous population with enhanced MHC-II antigen presentation [28,29]. However, a recent study has shown that cDC1 DCs are also capable of activating CD4+ T cells. In this study, they selectively removed MHC-II from cDC1 DCs, and this resulted in a reduction in CD4+ T cell-mediated responses [30]. A recent model suggests that CD4+ T cells are first primed by cDC2 and then reconnect with cDC1, to empower CD4+ T cells and enhance CD8+ T cell responses [31].

**Figure 1:**
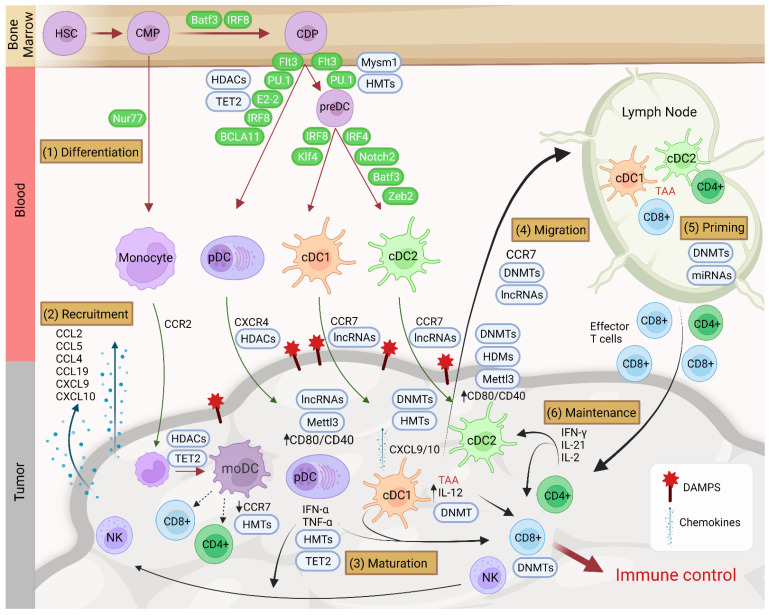
(1) Dendritic cells are originated from hematopoietic stem cells (HSC), which differentiate (represented with red arrows) into common myeloid progenitor (MDP) in the bone marrow. MDP can originate monocytes under the regulation of TF Nur77 or differentiate into common dendritic progenitor (CDP) under the TFs Batf3 and IRF8. Conventional type 1 DC (cDC1), conventional type 2 DC (cDC2), and plasmacytoid DC (pDC) subsets come from CDPs, under the influence of critical TFs and growth factors (shown next to each red arrow). (2) Under the expression of damage-associated molecular patterns (DAMPs) and chemokines like CCL2, CCL5 or CCL19 from the tumor the different DCs and monocytes are recruited in the tumor (represented with green arrows). This recruitment is guided by the expression of CCR2, CXCR4 and CCR7 in myeloid cells. (3) Once in the TME, DCs mature. This involves the upregulation of CD80/CD40 and the release of interleukin-12 (IL-12). In the case of cDC1, maturation processes also involve the expression of additional chemokines like CXCL9/10. Monocytes can further differentiate into monocyte-derived DCs (moDCs) in the tumor conditions. These cells have low expression of CCR7, which reduce their migration capacity. However, these cells are capable of activating CD4+ and CD8+ T cells within the tumor. (4) After tumor associated-antigen recognition (TAA), DCs migrate (represented with black arrows) to lymph nodes and (5) prime naïve CD4+ and CD8+ T cells. (6) After T cell activation, T cells migrate to the tumor and a constant cross-talk is produced between DCs and T cells to produce a correct immune control and maintenance. This maintenance consists in the expression of cytokines like interferon-α (IFN-α), tumor necrosis factor (TNF)-α from pDC or IL-12 from cDC that, in turn, is amplified by T- and natural killer (NK) cell–derived cytokines such as IFN-γ, IL-2 and IL-21. NKs can also be implicated in the release of chemokines, which will attract more DCs. During all these processes different epigenetic enzymes are implicated like ten-eleven translocation (TET)-2, DNA methyltransferases (DNMTs), histone deacetylases (HDACs), histone methyltransferases (HMTs), long noncoding RNAs (lncRNAs) or Mysm1 and the RNA methyltransferase Mettl3, represented in blue circles. Created with BioRender.com (accessed on 24 January 2022).

DC subsets are classified by their origin and function; they also differ with respect to the set of specific transcription factors (TFs) required for their development. Interferon regulatory factor 8 (IRF8) and IRF4 are necessary for the development of cDC1 and cDC2, respectively [32,33,34,35], accompanied by increased DNA accessibility [36]. These factors act in collaboration with PU.1, ID2, E2-2, ZEB2, Flt3 and BATF3, which have different degrees of specificity for DCs [29,33] (see Figure 1). For instance, the combination of whole-genome mapping of PU.1 binding and gene expression analysis has revealed a key role for this TF in maintaining cDC identity by inducing the transcriptional regulator DC-SCRIPT [37]. In this regard, the coordinated participation of transcription factors and histone modifications is critical. A good example of this is histone H2A deubiquitinase Mysm1, which mediates PU.1 recruitment in cDC differentiation [38]. Analysis of PU.1 and H3K4me1 shows an increasing overlap of PU.1 binding and H3K4me1 during cDCs differentiation [39].

Another DC subtype is exemplified by plasmacytoid DCs (pDCs), which differentiate from both the common dendritic cell precursor (CDP) and lymphoid progenitors [40,41]. Similar to cDCs, pDC express cytokine receptor Flt3 and are strictly dependent on its ligand Flt3L for their development [42]. Human pDCs were customarily defined as being those that express CD123, CD303 (BDCA2), CD304 (BDCA4), and immunoglobulin-like transcript 7 (ILT7) [43]. pDCs are mainly found circulating in the peripheral blood, however, are also present in peripheral organs. They have a characteristic surface phenotype and morphology that includes a highly developed secretory compartment [44]. This DC subset requires high levels of expression of IRF8, TCF4 (also known as E2-2) and BCL11A for their development, functional specification, and maintenance [45]. The TF E2-2 is essential for maintaining pDC identity as the loss of E2-2 in mouse pDCs causes them to differentiate into cDCs [46]. Some studies have observed that IRF8 does not play a key role in regulating pDC functions, although it is important for their development [47]. HDACs may also influence the differentiation of this subtype since, for example, the inhibition of HDAC reduces the expression of PU.1 and suppresses the recruitment of PU.1 to FLT3 and IRF8, which are fundamental TFs for the differentiation of pDCs [48].

Inflammatory conditions can lead to the recruitment of monocytes from blood, in a CC-chemokine receptor 2 (CCR2) dependent manner, and prompt them to differentiate to monocyte-derived DCs (moDCs) in peripheral tissues [49]. Mildner and colleagues regarded this subset as activated effector monocytes rather than cDC-like cells [50], on the basis of the pronounced proinflammatory signature. These cells have a similar phenotype to cDC2, however, can be distinguished by the absence of expression of CD26 [51]. Another special characteristic is the fact that they do not need the growth factor Flt3L for their development and they express low levels of Zbtb46, which are specific to the development of the other DC subsets [52,53]. This DC subset has been extensively studied using an in vitro model consisting of monocytes differentiating to DC-like cells in the presence of granulocyte-macrophage colony-stimulating factor (GM-CSF) and IL-4 [54]. Our group demonstrated that DNA demethylation plays a fundamental role in the differentiation from monocytes to moDCs, which depends on the JAK3-STAT6 pathway and TET2 [55]. Another example of epigenetic control in relation to DC differentiation comes from a study, in which the differentiation of monocytes to DC was blocked by pharmacological inhibition of HDACs [56].

## 4. Recruitment of Dendritic Cells in the TME and Involvement of Epigenetics

Analysis of the TME in patients with a variety of solid tumors has revealed immune cell infiltration, with two major phenotypes, based on the presence of infiltrating T cells: one has a broad chemokine profile and a type I interferon signature, while the other lacks this T cell-inflamed phenotype [57]. Not only solid tumors present immune cell infiltration. In fact, tumor-draining lymph nodes (TDLNs) are typically comprised of distinct subsets of immune cells [28]. Single-cell RNA sequencing studies have revealed a great complexity of immune cells in the TME [58,59,60]. Myeloid cells are a key cellular component among the immune cells that infiltrate into tumors and play important roles in modulating tumor inflammation and angiogenesis [61]. Immature DCs can be recruited into the TME, attracted by tumor-secreted factors such as CCL2, CCL20/MIP3a, CCL25, CCL5, CXCL12, CXCL1, and CXCL5, while mature DCs reside in areas surrounding the tumor [2,62,63]. The expression of the C-C chemokine receptor 7 (CCR7), the chemokine receptor for CCL19/CCL21, by DCs is important to facilitate their trafficking between the lymph nodes and the tumor [64,65]. Tumor-derived liver X-α receptor (LXRα) agonists in the TME can affect CCR7 expression in DCs since inhibition of LXRα increases protection against tumor growth [66].

Some specific treatments, like low-doses of CpG-B and GM-CSF, could result in the accumulation of cDC1 in the lymph nodes. An in vivo study has demonstrated that these treatments lead to the accumulation of cDC1 cells and recruitment in the lymph node by secreting type I interferon (IFN) [67]. It has been shown that non-coding RNAs, specifically lncRNAs, expressed exclusively in human cDCs, play a fundamental role in the differentiation and activation of DCs by directly binding to cytoplasmic signal transducer and activator of transcription 3 (STAT3) [68]. According to a recent study, lncRNAs may also affect DC migration, as they found that lnc-Dpf3 binds directly to the transcription factor HIF-1α and suppresses the HIF-1α-dependent transcription of the glycolytic gene Ldha, thereby inhibiting the glycolytic metabolism of DC and its migratory capacity [69].

pDCs are recruited to the specific target tissues from the bone marrow by the chemokine CXCR4. The NAD+ dependent deacetylase Sirtuin 6 (SIRT6) helps CXCR4+ DCs migrate to the afferent lymph nodes through its effects on histone H3K9 acetylation [70]. For moDCs, CCR2 seems to be necessary to ensure the correct migration of monocytes from blood to inflammatory tissue as there are fewer cells of this type in the inflammatory tissues of CCR2–/– mice [71]. After differentiation, moDCs display a limited capacity to migrate to the lymph node [72,73], which is related with the low expression of ccr7. This is epigenetically regulated as significant differences between migratory cDCs and non-migratory moDCs have been noted in the repressive levels of histone H3K27me3 in the locus of the ccr7 gene [74].

The TME contains a set of factors that inhibit the infiltration of DCs and reduce their immunosuppressive activity. Among them, a low level of expression of the chemokine CCL4 by tumor cells reduces the degree of DC infiltration [75]. Tumors can also limit DC infiltration indirectly by decreasing the viability of natural killer cells (NKs), which produce CCL5 [76]. The extent of infiltration is influenced by changes in DCs and possibly by DNA methylation changes in other infiltrated cells. For instance, Shi et al. reported a strong interaction between DNMTs and immune genes associated with the infiltration of neutrophils and DCs in colorectal carcinoma (CRC), suggesting that the TME was largely influenced by the methylation of related genes like ALOX5AP and CSF3R [77].

Cancer cells use a variety of epigenetically regulated pathways to avoid the immune system, which gives rise to a low level of migration of DCs. These include the downregulation of tumor-associated antigens (TAA), the loss of the antigen processing and presentation machinery, and the expression of Programmed Cell Death Protein 1 (PD-1) [78]. PD-1 is essential for inhibiting immune responses and promoting self-tolerance by modulating the activity of T-cells, activating apoptosis in antigen-specific T cells, and inhibiting it in regulatory T cells [79].

In general, the conditions found in the TME can promote, or not, the recruitment of certain subtypes of DCs, whereupon they can exercise a variety of functions (see Figure 1).

## 5. Functions of Dendritic Cells in the TME

Several studies have demonstrated the crucial role of DCs in anti-tumor responses [65,80,81,82]. One of the main functions of DCs in cancer immunity is the acquisition, processing and cross-presentation of TAAs, as well as the release of co-stimulatory and soluble factors to increase T cell response. In the TME, cancer cells induce expression of MHC-II, CD40, CD80, and CD86 on DCs, together with the release of inflammatory cytokines like IL-12, type I IFN, IL-1β, IL-6 and tumor necrosis factor (TNF) [83]. However, tumor cells can silence antigenic genes related to antigen presentation in DCs using epigenetic mechanisms that in turn impact antigen presentation by DCs [84,85,86,87].

One of the most efficient functions of DCs within the TME is the expression of IL-12, which polarizes naïve CD4+ T cells to T helper 1 (Th1), which enables them to express IFNα, and prime CD8+ T cells [88]. CD4+ T cells also help eliminate tumor cells through the production of IL-21 and IL-2, which are needed to adequately establish long-term memory T cells [89,90]. At the same time, IFN-α production increases the production of IL-12 by cDC1 and potentiates their antigen cross-presentation. This crosstalk proves to be even more complex since NKs and CD8+ T cells produce several factors that promote the recruitment of more cDC1 [91]. DCs also generate type I IFN, for example, when stimulated by anti-PD-1, a cancer immune therapy. In this context, stimulation of antitumor T cells by anti-PD-1 is not direct, but instead involves T cell:DC crosstalk, which is licensed by INF-γ and IL-12 [92]. Interferon expression is also epigenetically regulated by HMT KMT3A (SETD2) through the methylation of Lys 525 of STAT1 [93].

Each specific DC subtype has a different function in the TME. The crucial role of DCs in cancer immune response has been additionally demonstrated as cancer patients with more infiltration of cDC1 have a lower incidence of metastasis in oral, head and neck tumors [94] This could be related to the association of tumor-infiltrated cDC1 with the abundance of CD8+ T cells in the TME [64,95]. In addition, the presence of cDC1 in the tumor is associated with a better immune response to tumors [81]. cDC1 can also secrete CXCL9 or CXCL10 in the TME, which attracts CXCR3+ effector cells other than T cells, such as NK cells and group 1 innate lymphoid cells (ILC1) [96]. CXCL9/10 expression is not constitutive in cDC1, and it requires the expression of type I IFN or IFN-γ by T cells. One study demonstrated that the combined inhibition of DNMT1-mediated DNA methylation and EZH2-mediated trimethylation of H3K27 in a murine ovarian cancer model increased tumor infiltration of effector T cells by restoring levels of CXCL9 and CXCL10 production by Th1 [97]. The expression of these chemokines is important for more cDCs to infiltrate the TME, however, their expression can be suppressed by epigenetic enzymes such as KMT6A (EZH2), one of the DNMTs [97].

Initially, it seemed that cDC1 was the only DC subset involved in tumor immunity, however a more extensive analysis revealed the presence of other DC subsets [98] and the limited prevalence of cDC1 in human tumors [99]. Additionally, CD8+ T cells are not the only lymphocyte population involved in tumor immunity, and CD4+ T cells are known to be required in many tumor models [31,100,101,102,103,104]. It has been shown that cDC1 are not capable of activating CD4+ T cells ex vivo [99], and that the antigenic presentation of cDC2 to CD4+ T cells regulates multiple aspects of tumor immune response. Duong and colleagues reported an activation state of infiltrated cDC2s that was characterized by the expression of IFN-stimulated genes (ISG + DCs) and the ability to acquire and present intact tumor-derived peptide MHC class I complexes. These ISG + DCs can activate CD8+ T cells and promote protective anti-tumor immunity in the absence of cDC1 [105]. The same researchers subsequently found that this ISG + DC gene signature could be detected in human tumors.

pDCs are recognized by their main function of producing high levels of IFN-α in response to viruses and pathogens [106]. In addition, pDCs promote both innate and adaptive immune responses through induction of NK cell migration, macrophage and dendritic cell maturation, T cell response and antigen presentation [107]. IFN expression in pDCs is influenced by HMTs. For example, H3K9me2 levels are correlated with IFN expression levels, and the inactivation of the lysine methyltransferase G9a, which is essential for generating H3K9me2, consequently inhibits IFN production [108]. Type I IFN expression may also be influenced by DNA methylation since its induction requires TET2-dependent DNA demethylation of the IRF8 gene in pDCs [109]. Some models of breast cancer in mice have shown that pDCs alone can kill tumor cells through the expression of TNF, as well as promoting the activation of NKs [110]. Furthermore, pDCs occur in ovarian cancers, where they are essential for immunosuppression through their expression of indoleamine 2,3-dioxygenase 1 (IDO1) and inducible T cell costimulatory ligand (ICOSL) [111]. In addition, the enrichment of a specific subset of pDCs expressing high levels of TNF receptor (TNFR) superfamily member OX40 (CD134) in the TME has been reported. These pDCs can be discriminated by their distinct immunostimulatory phenotype, cytolytic function, and ability to synergize with cDCs to generate powerful tumor antigen-specific CD8+ T cell responses [112], thereby revealing another important facet of the involvement of pDCs in the TME. However, unlike with cDCs, tumor infiltration of pDCs is correlated with a poor prognosis in cancer patients. This is mainly due to pDC accumulation being associated with an increase in regulatory T cells (Tregs), which are naïve CD4+ T cells activated in the presence of transforming growth factor (TGF)-β and/or IL10 that have an immunosuppressive phenotype, resulting in decreased overall survival of the patients [113].

Once monocytes have reached the TME and have correctly differentiated to moDCs, they are capable of antigen presentation. Several studies have reported the presence of moDCs in the TME [82,114]. moDCs can also be found in the drainage from lymph nodes of mice with tumors that were treated locally with a combination of monosodium urate crystals and Mycobacterium smegmatis [114]. It has been established that moDCs are essential for CD8+ T cell activation and antitumor responses following local immunotherapy [114]. Even though different studies have also demonstrated the ability of these cells to present to CD4+ T cells [115]. Furthermore, moDCs differentiated for eight days in culture expressed CD141 and were able to capture dead cells and became mature when stimulated with TLR3 [116]. Finally, CD4+ and CD8+ T cells can be stimulated simultaneously if moDCs express MHC [117], although moDCs are scarce in lymph nodes [72,118]. The presence of this cell type in tumors is positively associated with cancer prognosis. Even though these cells may be present in tumors, their proportions are very low, and there is evidence that some tumors appear to exclude them actively [81] (see Figure 1).

## 6. Epigenetic Impact of the TME on Dendritic Cells

DC subtypes have the potential to promote correct immune responses. However, the TME contains immunosuppressive factors that limit the immunostimulatory properties of DCs. In the TME, tumor and immune cells acquire different characteristics that allow them to produce cytokines, chemokines, and growth factors. In cancer cells, a general activation of signaling pathways such as mitogen-activated protein kinase (MAPK), phosphoinositide 3-kinase (PI3K), STAT3, and nuclear factor κB (NF-kB) pathways happens, whereupon they promote the expression of IL-6, IL-10, GM-CSF and TGF-β, among others. This may help some of the infiltrated immune cells, especially DCs and NK cells, to lose their ability to present antigens and their cytotoxic function, respectively.

Various epigenetic modifications play a fundamental role in the differentiation and activation of DCs [4]. Together with the factors that are released by cancer cells, epigenetics may also have a main role in the transition to a tolerogenic phenotype of DCs. The relevance of epigenetics is exemplified by the pharmacological inhibitors of HDACs that block monocyte-to-DC differentiation, giving rise to a less immunogenic phenotype [56]. It has also been shown that TET2 represses the expression of the pro-inflammatory cytokine IL-6 by recruiting HDAC2 in order to resolve inflammation in innate myeloid cells [119]. Furthermore, the aberrant expression of miRNAs such as miR-22, miR-146a, and miR-146b inhibits the maturation and antigen presentation function of DCs, impairing their antitumor activity [120].

IL-10 expression in the tumor, which is mainly produced by macrophages [121], reduces the expression of MHC-II and CD40 in DCs [122], affecting their capacity to mature and to present antigens and this is epigenetically regulated by DNMTs and HDACs [85,86]. In this regard, repression in mature DCs of CIITA, a protein that acts as a positive regulator of MHC-II, is known to involve changes in histone acetylation across the whole gene locus and specific binding of positive regulatory domain 1 (PRDM1) to the promoters [123]. The expression of IL-10, together with GM-CSF, in the TME markedly increases the acetylation of histones H3 and H4 at the CIITA gene type I promoter locus during DC differentiation [124]. Recent studies have demonstrated that, under IL-10 expression, HDAC3, a class I HDAC, regulates the expression of proinflammatory cytokine like IL-12 in alveolar macrophages [125,126]. At the same time, HDAC11 negatively regulates the expression of the IL-10 gene in DCs in humans and mice [127].

IL-6 overproduction in the TME is associated with a functional defect in DCs in cancer patients [128]. In a different context, we have recently associated this cytokine with the acquisition of a tolerogenic phenotype. Specifically, vitamin D3 induces the in vitro acquisition of specific DNA demethylation and expression changes in DCs associated with tolerogenesis, and this is associated with direct vitamin D receptor (VDR) binding to genomic sites and the direct recruitment of TET2. This acquired tolerogenesis can be reverted by inhibiting JAK2/pSTAT3 [129].

Tumor-derived gangliosides and prostaglandin E2 (PGE2) are also produced in the TME, thereby altering the differentiation of cDCs and monocytes [130]. The expression of PGE2 by cancer cells also impairs the survival of NK cells by inhibiting the production of cytokines that attract cDC1 [76]. For instance, PGE2 levels are elevated in patients with colon cancer and are correlated with tumor size and patient survival [131], and are responsible for the reduced differentiation of DCs [132]. Another study also showed that PGE2 induces the upregulation of DNMTs and DNA hypermethylation of several genes [133].

More examples of secreted factors affecting the maturation and function of DCs, leading to the acquisition of a tolerogenic phenotype, include vascular endothelial growth factor (VEGF) and TGF-β, which inhibit normal DC maturation [134], and RANKL, a TNF family member that downregulates and upregulates the expression of IL-12 and IL-10, respectively [135]. VEGF is a protein responsible for the formation of tumor neovasculature and for tumor development [136,137]. This factor regulates DC migration and targeting by recruiting immature myeloid cells and immature DCs from the bone marrow [138]. After the inhibition of VEGF receptor 2, an increased in cDC2 infiltration occurs, together with increased production of IL-1β and IL-6, showing the relevance of this molecule in inhibiting DC infiltration and action in the TME [139].

TGF-β expressed by the tumor cells lowers the expression of DCs maturation markers like CD83, CD80, CD86, and MHC II molecules [140], and inhibits the expression of pro-inflammatory cytokines that induce the maturation of DCs, such as TNF-α, IL-1, IL-12, and IFN-α, while promoting the release of regulatory cytokines, including TGF-β itself [113,141]. TGF-β induces changes in histone H3K4me3 and H3K27me3 levels that cause the upregulation of costimulatory molecules and cytokines/chemokines and, in turn, the downregulation of differentiation markers [142].

Another study showed that the treatment of cell lines with two pro-inflammatory mediators found in TME, nitric oxide and IL-1β, increases the activity of DNMTs and leads to hypermethylation of a vast number of CpG islands [143]. DNA methylation can additionally silence endogenous retroviruses (ERVs), thereby activating the MDA5 pattern-recognition receptor, which normally detects viral infection by recognizing double-stranded (ds) viral RNAs. MDA5 induces signaling cascades that result in the secretion of type I interferon. HDAC and H3K4me1/2 have similar roles in ERV suppression and ERV-induced activation of the interferon pathway [144]. The cytokine-2 suppressor protein (SOCS2), a conserved program transcript, is another factor found in the TME. In primary melanoma, SOCS2 is expressed by mononuclear phagocytes that infiltrate these cells and is induced by IFN-γ. SOCS2 limits adaptive anti-tumor immunity and DC-based T-cell priming in vivo, indicating a critical regulatory role [145].

Other metabolites of the TME, such as lactic acid or reactive oxygen species (ROS), can reduce DC function. Lactic acid is a metabolic product that alters the differentiation and activation of moDCs, for example, by reducing the expression of IL-12 [146]. Several studies have shown that the production of ROS in the TME is associated with epigenetic changes. In one such study, treatment of a colorectal cancer cell line with hydrogen peroxide induced hypermethylation and subsequent silencing of potential tumor suppressor genes such as RUNX3 and CDX1 [147,148]. In another, oxidative damage was found to induce formation and relocalization of a silencing complex, which might explain cancer-specific aberrant DNA methylation and transcriptional silencing [149].

The production of factors released into the TME, such as VEGF, PGE2, and GM-CSF, pro-inflammatory cytokines such as IL-1β, IL-6, and TNF, and the peptides S100A8 and S100A9, can influence the differentiation of monocytes that arrive in the TME, where they accumulate and bestow a powerful immunosuppressive capacity, which affects their differentiation into moDCs [150].These cells are typically defined as myeloid-derived suppressor cells (MDSCs) [151], and may serve as a protective mechanism to prevent excessive tissue damage caused by unresolved immune responses [152]. MDSCs are a heterogeneous population of immature myeloid cells, characterized by the absence of surface markers associated with fully differentiated myeloid cells and by their morphological resemblance to granulocytic and monocytic cells [152]. These cells participate in many aspects of tumor progression, including immune evasion, angiogenesis, pre-metastatic niche formation, and epithelial-mesenchymal transition (EMT). It has been demonstrated that artificial TMEs, made with organotypic skin melanoma cultures (OMCs), prompts the transformation of normal immunostimulatory cDC2s into CD141+ DCs, with a phenotype matching their in vivo counterparts, and an impaired ability to stimulate T-cell proliferation, which is phenotypically similar to MDSCs [153].

MDSCs extracted from tumors produce secondary products such as ARGI, inducible nitric oxide synthase (iNOS), and ROS, that suppress the antitumor T response [154,155,156]. Additionally, it has been recently described that HMT SETD1B, which methylates histone H3 at Lys4, is responsible for iNOS upregulation in MDSCs [157]. Furthermore, pathways involved in cell trafficking and immunosuppression, including Wnt signaling, IL-6, and MAPK, are upregulated in MDSCs [158]. MDSCs also participate in tumor immune tolerance by generating Tregs through the secretion of TGF-β and IL-10 [159]. Cancer cells can also change the epigenome of MDSCs with respect to the expression of IL-6, whereby Krüppel-like factor 4, a transcription factor with zinc fingers, regulates the production of IL-6 in DCs through the acetylation of histones [160], thereby modifying the pattern of Treg accumulation.

Tetrahydrocannabinol, an exogenous cannabinoid, mediates epigenetic changes to promote MDSC differentiation and function, by a process in which S100A8 is closely involved [161]. miRNAs are other epigenetic marks that can regulate differentiation to MDSCs. For instance, the expression of STAT3 in MDSCs is modulated by miR-17-5p and miR-20a, which are usually negatively regulated [162].

Furthermore, our group has shown that the generation of monocytic MDSCs mediated by PGE2 depends on the upregulation of DNMT3A [163]. Comparison of the MDSC and DC DNA methylomes reveals specific gains in DNA methylation and repression of immunogenic-associated genes in MDSCs. Downregulation of DNMT3A in MDSCs abrogates the specific MDCS-specific hypermethylation and abolishes its immunosuppressive activity [163]. HDAC11 has been shown to regulate IL-10 gene expression in myeloid cells, [164]. This was demonstrated as tumor-bearing HDAC11-knockout mice presented an increased number of MDSCs compared to wild-type (WT) tumor-bearing control mice [164]. Finally, the use of DNMT3A-specific siRNAs can also restore the suppressor phenotype of MDSCs, demonstrating the influence of methylation on the acquisition of an immunosuppressed phenotype in a tumor environment [163].

## 7. Epigenetic Modifications in Dendritic Cells in Cancer Immunotherapy

DCs have properties that make them good candidates for generating tumor vaccines. Among the mechanisms most commonly used to produce cancer vaccines are the exposure of DCs to TAAs from cancer cell lysates [165]. Sipuleucel-T (Provenge) is the only DC-based vaccine to have been approved for use so far [166]. It consists of autologous blood DCs loaded with a recombinant fusion TAA composed of prostatic acid phosphatase and GM-CSF.

It might be possible to exploit the knowledge acquired regarding the effects of tumor cells on the epigenetic profiles of DCs to improve them for use in tumor immunotherapy. One of the most important properties of DCs that enables them to exhibit an anti-tumorigenic phenotype is their migration capacity, which arises from the expression of the CCR7 gene, along with the expression of IL-12. As explained earlier, cDC1 plays an integral role in tumor immunity and is a promising cell type for the development of DC-based vaccines. However, no clinical trials have used ex vivo-derived cDC1 for adaptive cell transfer, largely as they are very scarce in blood, accounting for fewer than 1% of PBMCs, and also as they are difficult to obtain ex vivo in such a way as to maintain their functional phenotype. The latter problem can be attributed to several factors, including the different patterns of antigen expression arising from the heterogeneity of the tumor, the low levels of tumor-infiltrated lymphocytes (TILs), and the evolution of different immunosuppressive mechanisms as the tumor develops [167].

**Figure 2:**
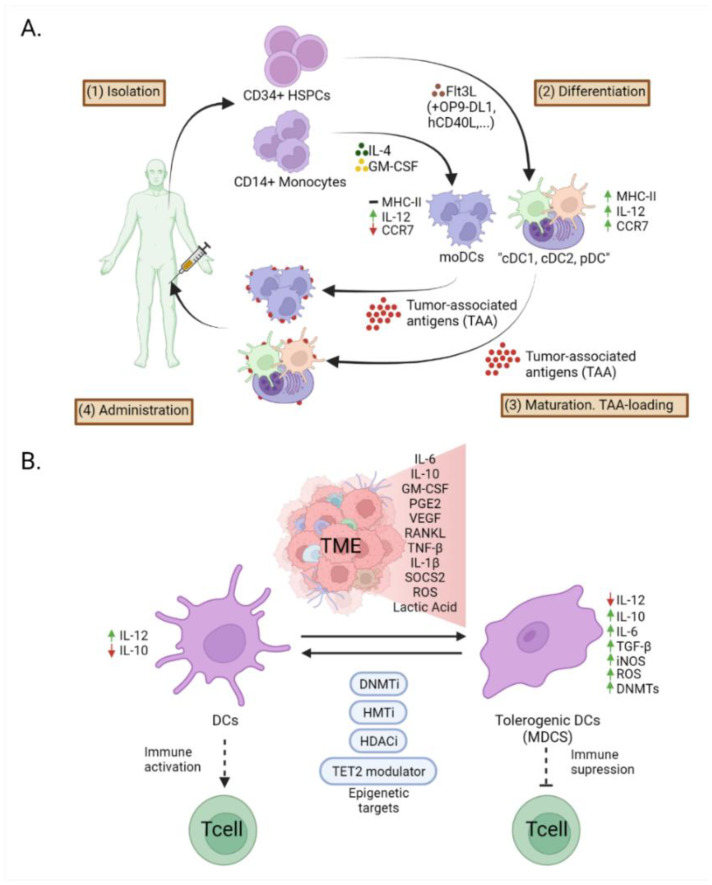
(**A**). Methods used for DC-based cancer vaccines. Black arrows indicate conventional manufacturing steps. CD34+ haematopoetic stem progenitor cells (HSPCs) are isolated from cancer patients and differentiated into and conventional type 1 DC (cDC1), conventional type 2 DC (cDC2), and plasmacytoid DC (pDC) like cells with Flt3L together with other to improve the resulting DCs. CD14+ monocytes are isolated and differentiated and monocyte-derived dendritic cells (moDCs) with IL-4 and GM-CSF. moDC presents low antigen presentation and low migration capacity, together with the expression of interleukine-12 (IL-12) and the cDC1, cDC2 and pDC like cells present better migration capacity, antigen presentation and produce cytokine like IL-12 and interferon (IFN)-α. DCs are then loaded with tumor-associated antigens (TAAs) and administrated again to the cancer patient. (**B**). Soluble factor and exosomes secreted in the tumor microenvironment (TME), including IL-6, IL-10, granulocyte-macrophage colony-stimulating factor (GM-CSF), prostaglandin E2 (PGE2), vascular endothelial growth factor (VEGF), RANKL, tumor necrosis factor (TNF)-β, IL-1β, cytokine-2 suppressor protein (SOCS2), reactive oxygen species (ROS) and lactic acid, dendritic cells acquire an immune tolerance phenotype. The resultant cells, present low expression of IL-12 but increase expression of IL-10, IL-6, TGF-β, nitric oxide synthase (iNOS), ROS and also present more expression of DNA methyltransferases (DNMTs). This process can be reverted by targeting epigenetic enzymes like ten-eleven translocation (TET)-2, DNA methyltransferases (DNMTs), histone deacetylases (HDACs), histone methyltransferases (HMTs). Created with BioRender.com (accessed on 24 January 2022).

Many attempts have been made to apply the use of cDC1 cells to design strategies to fight tumors. One of the strategies used involves differentiating precursor cells, such as those in BM culture treated with Flt3L, to develop a mixture of cells that resemble pDCs, cDC1, and cDC2 DCs [168]. A consideration of epigenetics is important for acquiring these DCs from BM, since stimulation with GM-CSF increases the expression of the Pdcd1lg2 gene, which is accompanied by increases in PU.1 binding and histone acetylation. The participation of PU.1, IRF4, and p300 in mouse splenic DCs has also been noted [169]. However, the DCs obtained do not express the typical cDC1 markers. Kirkling et al. treated monolayer murine BM precursor cells for three days with FLT3L, OP9-DL1 resulting in cells expressing typical cDC1 markers such as CD103, CD24, DEC205, and CD8a. The transcriptome of the derived cells has a similar pattern to that of cDC1 purified from the spleen. These cells led to better vaccination outcomes since, when loaded with ovalbumin, they improve survival. These results may be partially attributed to the better migration of the resulting cDC1 to the lymphatic ganglia, as these also have a higher expression level of the ccr7 gene, the encoding protein of which guides DCs to the lymph nodes [170,171] (Figure 2A).

In recent years, epigenetic therapies have been considered a promising option in the fight against cancer, where they can be used to improve cancer vaccines. For example, during the activation of DCs with LPS, HDMs such as KDM6B (JMJD3) and KDM4D (JMJD2D) eliminate the repressive marks H3K27me3 and H3K9me3, thereby regulating pro-inflammatory genes and stimulating inflammation. [172,173]. In this fashion, the intratumoral activation of HMT KDM6B or HDM KDM4D could be considered important for promoting local DC activity. Another example is the use of EPZ004777, which reduces the levels of H3K79me2, to improve the function of DCs in TME. This causes a drop in the level of expression of the Forkhead box M1 transcription factor (FOXM1), which is a proliferation-associated transcription factor involved in tumorigenesis through the transcriptional regulation of its target genes in various cells, including DCs. In the case of pancreatic and colon cancers, FOXM1 has an immunosuppressive role through impaired DC maturation [174]. One study reported that the low-dose combination of two FDA-approved epipharmaceuticals, the DNMT inhibitor (DNMTi) 5-azacytidine and the HDAC inhibitor (HDACi) romidepsin, with IFN-α limits the aggressiveness of colorectal cancer stem and metastatic cells in vivo and triggers immunogenic cell death signals that stimulate DCs function and increase their migratory capacity [175].

One of the most common strategies aims to improve migration capacity through the expression of ccr7. This emerged from a study, in which the overexpression of CCR7 improved accumulation in the lymph nodes that drain tumors [176]. An attempt has recently been made to improve the bone marrow-derived DCs (BM-DCs) by targeting β2-integrin. The latter are a family of heterodimeric adhesion receptors with a common β2 chain (CD18). β2-integrins are expressed by leukocytes and are involved in immune synapse formation, phagocytosis, and adhesion. BM-DCs that express dysfunctional β2-integrin have enhanced tumor rejection capabilities in B16.OVA and B16-F10 melanoma models and higher levels of expression of CD86, Il12, ccr7, and Fscn1, which are indicative of improved co-stimulation and migration capacity. These changes were associated with epigenetic changes such as overall increases in both chromatin accessibility and levels of histone H3K4me3/H3K27me3 methylation. These changes were in turn related to transcription factors such as Ikaros and RelA [177]. Deletion of microRNA-155 (miR-155) in BM-derived moDCs in mice increases H3K27me3 levels, leading to transcriptional repression of CCR7 [178]. This also impairs DC maturation and IL-12 secretion [178], with miR-155 proving to be a possible target with which to improve moDC therapies. However, the role of miR-155 has not been studied in cDC1; addressing this would open a new area of study. Finally, it has recently been reported that once cDCs have performed antigen presentation, they increase the expression of ccr7 and their capacity to migrate to the lymph nodes, thereby also increasing their antigenic capacity [179]. The same study also noted that the upregulated genes in these so-called post-synaptic DCs were epigenetically regulated as their accessibility increased. The authors describe a previously unknown population of DCs whose transcriptomics, epigenomics, and migratory capacity change in response to their cognate contact with T cells [179].

Another possible strategy to improve DC-based vaccines is to improve their antigen presentation capacity. In this regard, it has been seen that vitamin C (VitC) modulates anticancer immune responses and, in turn, is a cofactor of TET2 [180]. In a recent study, it was shown that VitC treatment not only enhances the cytotoxic activity of adoptively transferred CD8+ T cells, but also cooperates with immune checkpoint therapy (ICT) in various types of cancer [181]. A possible field of study would be to study the effects of VitC on DCs since this could improve the ability of DCs to present tumor antigens to CD8+ T cells through the specific demethylation of certain genes.

As previously mentioned, the biggest concern is the scarcity of cDCs in BM-derived cells. In this context, a therapy based on moDCs is a promising alternative. One property that makes the moDC type special is that GM-CSF-induced moDCs can also be used as a vaccine when they are matured ex vivo with CD40 ligand, IFN-γ, and/or TLR agonists. moDCs have a limited capacity to cross-present antigens or to migrate to the lymph nodes. Some phase III clinical trials of the use of moDCs in treating uveal melanoma (NCT01983748), castration-resistant prostate cancer (NCT02111577), and metastatic colorectal cancer (NCT02503150), are currently underway. Preliminary results from a large trial (NCT00045968) of a moDC-based vaccine loaded with autologous tumor lysates have shown that this treatment is feasible and safe in glioblastoma patients. Similarly, intratumoral and intranodal administration is known to induce an equivalent immune response and efficacy in breast cancer patients treated with moDCs [182] (Figure 2A).

Finally, epigenetics can also be used to improve the immune response of cells that are already affected by TME-like MDSCs, which are a potential target for cancer therapy. However, the low TLR-mediated activation capacity of MDSCs makes their use in immunotherapy challenging. Several clinical trials that use MDSCs as a target in a variety of cancer types including leukemia, breast cancer, melanoma, and glioblastoma, are underway. These therapies involve targets like Arg1, iNOS, STAT3, CD36, and CXCR2 [159]. There are also therapies undergoing phase I/II clinical trials targeting MDSCs that indirectly inhibit HDACs by using atezolizumab (NCT03024437). HAT CBP/P300 promotes the suppressor function of MDSCs by increasing the levels of H3K27Ac in promoters and enhancers of proto-tumor genes. Its inhibition hampers its suppressive activity in the colon carcinoma model [183]. In addition, HDACi entinostat class I is reported to have antitumoral properties since it has been shown to neutralize MDSCs through epigenetic reprogramming in mouse models of breast, pancreatic, and renal cell cancer [184,185]. Liu et al. reported a deregulated miR-148a/DNMT1/SOCS1 axis as being a unique mechanism for stimulating buffered TLR in MDSCs. They determined that miR-148a was elevated in MDCS polyinosinic-polycytidylic acid (poly I: C) or that DC maturation was induced by LPS by the direct suppression of the DNMT1 gene, which consequently led to hypomethylation and thereby upregulation of SOCS1, the suppressor of TLR signaling [186]. Finally, Orillion et al. found that the application of the HDACi entinostat reduces the levels of MDSC-associated chemoattractants and MDSC suppressive activity, and enhanced the efficacy of anti-PD-1 therapy [184] (Figure 2B).

Although several studies have demonstrated the value of DC and MDSC epigenetics in improving cancer vaccines, this is still a matter that warrants further exploration.

## 8. Conclusions

DCs play a fundamental role in the response to tumors. However, the TME produces a number of factors that can modulate DCs immune response, thereby acquiring an immunosuppressive phenotype that allows tumor growth. Recent evidence has shown the fundamental role of epigenetics in the regulation of DCs, both in their differentiation and in their recruitment in the TME, as well as in the response against cancer cells in the TME. It is important to note that there are different types of DCs, including cDC1, cDC2, pDC and moDCs, and each one has a different role in the immune response against the tumor, either by migrating to the lymph node and activating the effective Tcells, or in the maintenance of the immune response within the tumor. Epigenetic regulation in the various DC subtypes by itself may elicit more robust antitumor immunity as an interventional approach. However, the different factors secreted by the tumor can cause these DCs to acquire a tolerogenic phenotype, allowing tumor growth. In this case, epigenetics is also playing a fundamental role in this transition. Therefore, a rational strategy to further increase immunotherapeutic efficacy is to combine certain epigenetic regulators to improve DC-based vaccines for cancer or drugs that reverse the tolerogenic phenotype acquired by DCs under the influence of the tumor microenvironment.
